# Prevention of Shivering Post Subarachnoid Block: Comparison between Different Doses of Intravenous Magnesium Sulphate

**DOI:** 10.3390/medicina58081046

**Published:** 2022-08-03

**Authors:** Ren Geng Low, Azarinah Izaham, Jaafar Md Zain, Nadia Md Nor, Hsueh Jing Low, Aliza Mohamad Yusof

**Affiliations:** Department of Anaesthesiology and Intensive Care, Universiti Kebangsaan Malaysia Medical Centre, Jalan Yaacob Latif, Bandar Tun Razak, Cheras, Kuala Lumpur 56000, Malaysia; rglow325@hotmail.com (R.G.L.); azaizaham@yahoo.com (A.I.); jaafar@ppukm.ukm.edu.my (J.M.Z.); nadiamn72@yahoo.com (N.M.N.); jing_my@yahoo.com (H.J.L.)

**Keywords:** prevention, shivering, subarachnoid block, magnesium sulphate

## Abstract

*Background and Objectives*: Shivering is a common complication of subarachnoid block (SAB). Magnesium sulphate has been proven to be effective in preventing shivering. The aim of this study was to compare the effectiveness and adverse effects in hemodynamic parameters between 50 mg/kg and 30 mg/kg of intravenous magnesium sulphate for prevention of shivering post-subarachnoid block. *Materials and Methods*: Eighty-six patients scheduled for surgery under SAB, aged between 18 to 65 years old with American Society of Anesthesiologists physical status I and II were randomised into two groups. Group A received a bolus of 50 mg/kg, while Group B received 30 mg/kg of intravenous magnesium sulphate, given over a 20 min duration following SAB. Shivering grade was recorded intraoperatively according to the Crossley and Mahajan shivering scale. Mean arterial pressure (MAP), heart rate, tympanic temperature, oxygen saturation and the use of vasopressors were recorded. *Results*: Forty-five percent of patients in Group A and 20% of patients in Group B did not exhibit shivering (*p*-value < 0.01). High-grade shivering was observed in 12.5% in Group A and 40% in Group B, respectively (*p*-value 0.02). The MAP trend was lower in Group B (*p*-value < 0.01), but the incidence of hypotension was not significant in both groups. The use of vasopressors was also similar between groups. Group B showed a lower oxygen saturation trend (*p*-value 0.04). The trends of heart rate and tympanic temperature were not significant in both groups. No patients had episodes of bradycardia or oxygen desaturation. *Conclusions:* In this study, intravenous magnesium sulphate 50 mg/kg is the lowest effective dose for prevention and treatment of high-grade shivering post-SAB without significant hemodynamic adverse events.

## 1. Introduction

Shivering is one of the most frequent and disturbing complications associated with subarachnoid block (SAB). Shivering is an involuntary, oscillatory muscular activity that augments metabolic heat production, which can be an unpleasant experience for patients, surgeons and anaesthetists [1]. The incidence of shivering is 40–70%, and most commonly occurs 20 min post-SAB [2,3,4,5]. 

The episode of shivering in anaesthesia is unfavourable because it can increase the metabolic rate up to four-fold, doubling or tripling the oxygen consumption and carbon dioxide production, which could subsequently lead to arterial hypoxaemia and lactic acidosis, especially in the elderly [6]. It can also trigger myocardial ischaemia and may cause the increase of intracranial and intraocular pressure [7]. Furthermore, shivering can interfere with monitoring of oxygen saturation (SpO₂), non-invasive blood pressure (NIBP) and electrocardiogram (ECG) [8].

Numerous studies have tested the efficacy of a large variety of drugs to prevent shivering in anaesthetised patients, but their cost is high or their safety is questionable. Sedative agents such as clonidine and midazolam may not be appropriate for recovery post-anaesthesia [1,6]. The hypertensive and tachycardiac effects of ketamine limits its use [6]. Pethidine has been shown to be one of the most effective drugs in managing shivering post-SAB, as it is the only opioid agonist at both μ and κ receptors, which are known to reduce the shivering threshold. However, pethidine may cause nausea and vomiting, and increases the risk of respiratory depression during and after anaesthesia with concurrent use of parenteral or neuraxial opioids [9]. Thus, the search continues for drugs with better safety profiles in managing perioperative shivering.

Magnesium sulphate is a non-competitive antagonist of N-methyl-D-aspartate (NMDA) receptors, and it is also a naturally occurring calcium antagonist. It has been used to increase onset and reduce requirement of muscle relaxants, to act as bronchodilator, neuroprotection or antiarrhythmic agent, and as an adjuvant analgesic to reduce opioids use in anaesthesia [10]. 

A recent systemic review and meta-analysis by H. Kawamikami et al. has confirmed that intravenous (IV) magnesium effectively reduces shivering, and the incidence of adverse effects is not significant with magnesium administration. In this study, the author classified IV magnesium greater than 60 mg/kg as the high-dose category and dosages less than 60 mg/kg as the low-dose category. It was concluded that both categories were effective in reducing the incidence of shivering in comparison with control group. They also concluded that an IV dose of more than 60 mg/kg of magnesium sulphate will not dose-dependently reduce the incidence of shivering, and for the low-dose category, they were unable to determine the minimal effective dose [11]. Hence in our study, we determine the lowest effective dose for prevention of post-SAB shivering by comparing the efficacy of IV magnesium sulphate at 50 mg/kg and 30 mg/kg.

## 2. Materials and Methods

This prospective, double blind, randomised control trial was approved by the Research Committee of Department of Anaesthesiology and Intensive Care, Universiti Kebangsaan Malaysia Medical Centre (UKMMC) and Medical Research & Ethics Committee, UKMMC (JEP-2020-304). Written informed consent was obtained from the patients recruited into the study.

Eighty-six patients aged 18 to 65 years old, American Society of Anaesthesiologists (ASA) I or II, who were scheduled for either elective or emergency non-obstetric surgery (orthopaedic, general surgery, vascular, urology and gynaecology) under SAB with estimated surgery time more than 30 min were recruited into the study. Patients who had known allergies to study drugs, cardiac arrhythmia, history of seizures, peripheral neurological disease or neuromuscular disease, or pre-operative body temperature less than 35 degree Celsius (°C) or more than 38 °C were excluded. Patients were randomised into two groups using a computer-generated code. Group A were given intravenous bolus infusion of magnesium sulphate 50 mg/kg, while Group B were given 30 mg/kg, which started immediately after SAB. Both doses were diluted in 50 ml normal saline and given over 20 min. Patients who required conversion to general anaesthesia, encountered massive blood transfusion during surgery or had a surgery time less than 30 min were dropped out from the study. 

Prior to induction, all patients fasted for at least six hours before surgery and no premedication was administered. A peripheral venous cannula of at least 20 gauge and above was inserted into the hand of patients on their admission to the hospital by the respective ward doctor. In the operating theatre, standard monitoring was applied to all patients including pulse oximeter, ECG and NIBP. All patients were given warmed IV co-loading crystalloid fluid at 10 mls/kg. Core body temperature was measured at the tympanic membrane using an infrared ear thermometer. The temperature of the operating room (OR) measured by wall thermometer was recorded at the beginning of surgery. The ambient OR temperature were maintained between 18 to 22 °C. Blood pressure (BP), mean arterial pressure (MAP), heart rate (HR), SpO₂ and tympanic temperature were measured prior to SAB (baseline) and immediately after spinal anaesthesia, which was taken as time 0, then at 5, 10, 15, 20, 25, 30 and 60 min, or until the end of the surgery. Post-operatively, these vital signs were recorded upon arrival and prior to discharge from recovery area.

Subarachnoid block was performed with the patients in the sitting position at L3/L4 or L4/L5 interspace, with a midline approach using a 27 G spinal needle under aseptic technique. The local anaesthetic agent given for SAB comprised of 0.5% hyperbaric bupivacaine 12.5 to 15 mg, with or without fentanyl 10 to 15 mcg. Patients were put in supine position immediately after SAB and patients were given the bolus infusion of the study drug over 20 min via syringe pump by the anaesthetist or anaesthesia medical officer in-charge, which was taken as time zero. Motor block was assessed 5 min after SAB using the modified Bromage scale and the sensory block was assessed using cold cotton wool. All patients were actively warmed using a forced air warming device, set at 38 °C. 

Intraoperatively, IV atropine 0.5 mg was administered if a patient’s heart rate fell below 50 beats per minute. Hypotension was treated with ephedrine 6 mg or phenylephrine 100 µg IV per bolus and with further IV infusion of crystalloid solution as required. Hypotension is defined as MAP of less than 60 mmHg or a decrease in MAP of more than 20% from baseline. Oxygen supplement was given if patient’s SpO₂ was less than 95%. These episodes of bradycardia, hypotension and oxygen desaturation were documented as the medication’s side effects. 

The grade of shivering was evaluated using a 5-point scale that has been validated by Crossley and Mahajan (grade 0 = no shivering, grade 1 = piloerection, vasoconstriction without visible muscular activity, grade 2 = visible muscular activity confined to one muscle group, grade 3 = visible muscular activity of more than one group, grade 4 = gross muscular activity involving the entire body). Shivering was considered significant when the patient suffered at least grade 3 shivering [12]. The grade of shivering was observed by an independent observer blinded to the dosage of the study drug throughout the surgery, and the highest shivering grade was documented from 15 min of IV magnesium sulphate infusion. A rescue dose of IV pethidine 25 mg was administered if shivering grades 3 or 4 persisted. Monitoring for vital signs and shivering was continued in the recovery room until the patient was discharged to the ward.

Data analysis was performed using SPSS for Windows version 25.0 (IBM Corp, Armonk, NY, USA). Results were presented as mean ± standard deviation, median (interquartile range) or frequency (percentages) where appropriate. For between-group analysis, an independent t test was used for normally distributed data. The qualitative data was analysed using the Chi square test. Continuous data between two groups was analysed using a two-way mixed ANOVA test. A *p* value < 0.05 was considered as statistically significant.

## 3. Results

Eighty-six patients were recruited into this study and randomly assigned into two groups. A total of six patients, three from each group, were dropped, making the total number 40 patients in each arm. The drop-outs were due to a failed block requiring conversion to general anaesthesia or surgery that was completed in less than thirty minutes. Table 1 demonstrates the demographic data. There were no significant differences in gender, age, discipline, ASA, weight, body mass index (BMI), duration of surgery and OR temperature between groups. 

Table 2 showed a significant difference of the incidence of the highest shivering grade that occurred between the groups (*p*-value < 0.01). Group A demonstrated a significantly higher incidence of absence of shivering (grade 0) in comparison with Group B. The incidence of patients with severe shivering (grade 3 and 4) was significantly higher in Group B compared to Group A. The incidence of mild shivering (grade 1 and 2) was not significant between groups. Patients in Group A required significantly lower rescue IV pethidine in comparison with Group B (10% versus 32.5%, *p*-value 0.026).

Table 3 shows the shivering grade comparison between groups starting at the 15th min of IV magnesium sulphate infusion. Patients in Group A had significant absence of shivering (grade 0) in comparison with Group B at the 25th, 30th and 60th min, in recovery bay and upon discharge to ward. Significantly less patients in Group A had severe shivering (grade 3 and 4) at the 25th, 30th and 60th min of IV magnesium sulphate infusion compared to Group B. None of the patients had severe shivering upon discharge to ward.

**Figure 1:**
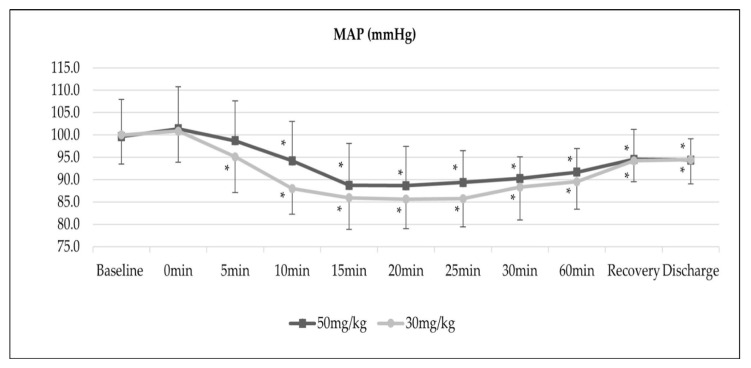
Trend of MAP, *p* = 0.008; * p < 0.05 (intragroup comparison with baseline MAP).

Figure 1 demonstrates the trend of MAP of both groups. The MAP trend was significantly lower in Group B compared to Group A (*p*-value < 0.01). A significant decrease in MAP between baseline at the 10th, 15th, 20th, 25th, 30th and 60th min, at recovery and during discharge was observed in both groups. 

**Figure 2:**
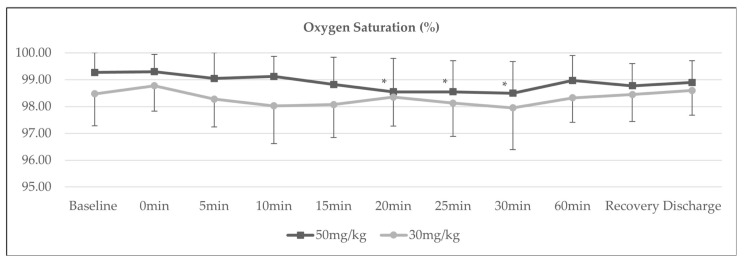
Trend of oxygen saturation, *p* = 0.040; * *p* < 0.05 (intragroup comparison with baseline MAP).

The trend of SpO_2_ over time was significantly lower in Group B compared to Group A (*p*-value 0.04), as shown in Figure 2. Group A demonstrated a significant reduction in oxygen saturation at the 20th, 25th and 30th min compared to baseline (*p*-value 0.02, 0.006 and 0.004, respectively).

**Figure 3:**
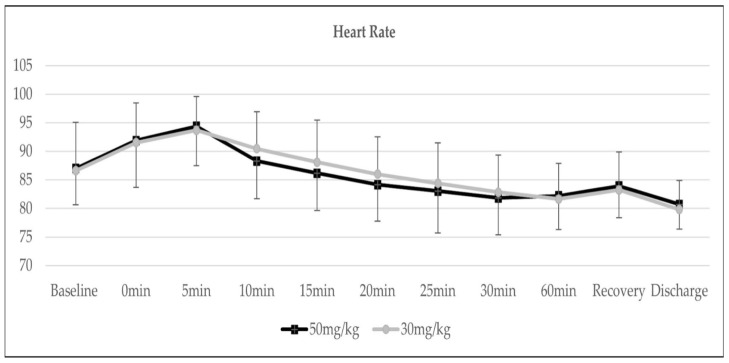
Trend of heart rate, *p* = 0.150.

**Figure 4:**
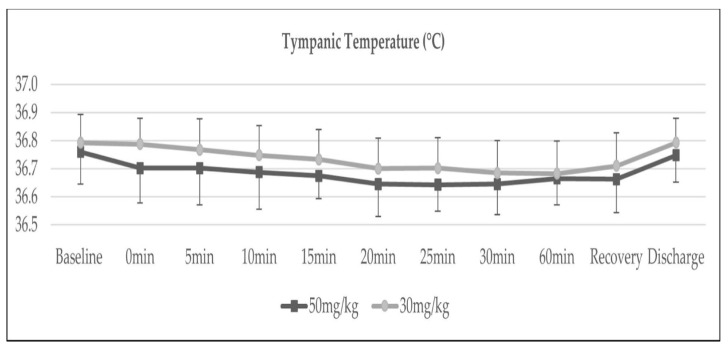
Trend of core temperature, *p* = 0.634.

The trends of heart rate and core temperature were insignificant between both groups, as demonstrated in Figure 3 and Figure 4.

The incidence of hypotension was not significant, with 25% in Group A and 10% observed in Group B. The mean doses for phenylephrine in both groups also were not significant: 150 µg ± 54.8 in Group A and 133.3 µg ± 57.7 in Group B. The mean requirement of ephedrine was comparable between Group A and Group B (8 mg ± 3.5 versus 6 mg ± 0). No episodes of oxygen desaturation or bradycardia were documented among the groups.

## 4. Discussion

Our study demonstrated that the IV magnesium sulphate 50 mg/kg bolus is able to prevent shivering post-SAB and lower the incidence of high-grade shivering. Gozdemir et al. demonstrated a significant reduction in post-spinal anaesthesia shivering in patients who underwent transurethral prostatectomy, where a bolus of IV magnesium sulphate 80 mg/kg followed by 2 gm/hour of infusion was compared with a placebo group (6.7% versus 66.7%, *p*-value < 0.01) [13]. Their incidence of shivering post-SAB is smaller compared with our study, which could be due to the higher bolus dose of IV magnesium sulphate. In a study by Ibrahim et al., the use of prophylactic IV bolus of magnesium sulphate 50 mg/kg followed with 2 mg/kg per hour of infusion versus saline in post-spinal anaesthesia also showed a lower incidence of high-grade shivering in the prophylactic group (15% versus 50%, *p*-value < 0.01), which is similar to our results [14]. Forty-percent of our patients that were prescribed with 30 mg/kg of IV magnesium sulphate had severe shivering, which corresponds with findings by Kasem at al. The authors also observed that the episodes of shivering were comparable when comparing 30 mg/kg of IV magnesium sulphate with IV pethidine 0.5 mg/kg (40% versus 20%), [15], which was different when a higher dose of IV magnesium was administered, as demonstrated by Elsonbaty et al. Elsonbaty reported that the incidence of shivering post-SAB was significantly reduced to 28% in magnesium group with a bolus of IV magnesium sulphate 50 mg/kg, followed by 0.5 mg/kg/min infusion, compared with IV pethidine 0.5 mg/kg [16]. These outcomes suggest that IV magnesium sulphate 50 mg/kg is the lowest effective dose in prevention of shivering and reduction of shivering grade in comparison with 30 mg/kg. We also observed that episodes of shivering occurred 25 min after SAB, which was recognised as the onset of shivering by Luggya et al. It was concluded that the mean time of shivering onset was between 15 to 25 min post-SAB [5]. 

It is known that the IV magnesium sulphate induces a dose-dependent decrease in blood pressure [17], and it was interesting to observe that the patients who received IV magnesium sulphate 30 mg/kg had a lower trend of blood pressure during the period of infusion. Sachidananda et al. demonstrated that the MAP trends of patients given an IV bolus of magnesium sulphate 30 mg/kg were significantly lower than the saline group, in contrast with Hwang et al. [18,19]. Hwang et al. demonstrated that although the patients who received 50 mg/kg of IV magnesium sulphate followed by 15 mg/kg/hour infusion post-SAB had a lower MAP, it was not significant compared to those receiving saline [19]. This finding is also similar to that of Zhong et al., who exhibited that the trend of systolic blood pressure in those prescribed with 50 mg/kg of IV magnesium sulphate versus saline was reduced, but comparable, except at the 15 min time-point of medication infusion [20]. The effect of a lower fluctuation of MAP in patients prescribed with a high dose of IV magnesium sulphate could be related to the relationship of stroke volume, cardiac output and peripheral resistance. It was found that the high dose of IV magnesium sulphate, which is taken as a dose of more than 60 mg/kg, reduces peripheral vascular resistance and causes z significant increase in the stroke volume; as a result, cardiac output as well as blood pressure is maintained [21]. England et al. conducted a study of the effect of 2 gm of IV magnesium chloride on cardiovascular function during cardiac surgery and they observed identical findings of increase in stroke volume [22]. 

Another intriguing finding in our study was that a brief reduction of oxygen saturation was documented between 20 to 30 min post-SAB in the 50 mg/kg group. While magnesium sulphate is a known bronchodilator and is particularly useful as a treatment to improve oxygenation for acute obstructive pulmonary disease, a decrease in oxygen saturation could be observed, and the effect appears to be transient in patients given IV magnesium sulphate [23,24]. 

As for the trend of core temperature changes across time, our study was unable to demonstrate significant findings. This is in contrast with the studies by Gozdemir et al. and Ibrahim et al.; both studies showed a significant reduction in core temperature in patients receiving IV magnesium sulphate [13,14]. The discrepancy in our results could be due to our methodology involving active warming of patients using a forced air warming device, blanket, warm co-loading fluids and maintenance of OT temperature between 18 to 22 °C, which eventually confounded the detection of hypothermia.

Despite the significant changes of MAP from the 10th min, which was occurred in both groups, the incidence of hypotension was similar. Ibrahim et al. reported similar incidences of hypotension—detected in 15% of patients—and it was not statistically significant when compared to the controlled saline group. The use of vasopressors, particularly ephedrine, was also shown to have no difference, resembling our observations [14].

We acknowledge that our study had limitations. The absence of a control group in our methodology could affects the interpretation of our results, and it should be addressed in future studies. Our results also cannot be extrapolated for obstetric and elderly patients more than 65 years-old, as the incidence of hypotension in patients who underwent Caesarean section prescribed with IV magnesium sulphate for post-SAB shivering prevention was greater [17]. Therefore, further studies are needed to confirm the optimum and safe dose of magnesium sulphate for the prevention of shivering post-SAB for obstetric as well as elderly populations.

## 5. Conclusions

In this study, intravenous magnesium sulphate 50 mg/kg was the lowest effective dose for prevention and treatment of high-grade shivering post-SAB, without significant hemodynamic adverse events.

## Figures and Tables

**Table 1:** Demographic data.

Variable	Group A (50 mg/kg)	Group B (30 mg/kg)	*p*-Value
	(n = 40)	(n = 40)	
**Gender**			
Male	26 (65.0)	25 (62.5)	0.816
Female	14 (35.0)	15 (37.5)	
**Age (years)**	40.9 ± 12.9	46.4 ± 12.8	0.948
**Discipline**			
Orthopaedic	21 (52.5)	25 (62.5)	0.267
Surgery	12 (30.0)	8 (20.0)	
Urology	6 (15.0)	3 (7.5)	
Gynaecology	1 (2.5)	4 (10.0)	
**ASA class**			
ASA I	18 (45.0)	15 (37.5)	0.496
ASA II	22 (55.0)	25 (62.5)	
**Weight (kg)**	71.1 ± 10.2	69.1 ± 9.5	0.653
**BMI (kg/m^2^)**	25.9 ± 2.8	26.2 ± 2.2	0.143
**Duration of Surgery (minutes)**	62.8 ± 7.9	64.2 ± 7.4	0.44
**OR Temperature (°C)**	19.3 ± 1.2	19.5 ± 1.1	0.624

Data are expressed as mean ± standard deviation or frequency (percentage).

**Table 2:** Highest shivering grade after 15 min of subarachnoid block.

Shivering Grade	Group A (n = 40)	Group B (n = 40)	*p*-Value	*p*-Value (Post-Hoc Test)
No shivering (grade 0)	18 (45.0)	8 (20.0)		0.016 *
Mild (grade 1–2)	17 (42.5)	16 (40.0)	0.008 *	0.82
Severe (grade 3–4)	5 (12.5)	16 (40.0)		0.002 *

Data are expressed as numbers (percentage); * statistically significant, *p* < 0.05.

**Table 3:** Shivering grade at every time post-SAB.

Time Point	Shivering Grade	Group A (n = 40)	Group B (n = 40)	*p*-Value	*p*-Value (Post-Hoc Test)
15th min	No shivering (grade 0)	33 (82.5)	36 (90.0)		-
	Mild (grade 1–2)	7 (17.5)	4 (10.0)	0.117	-
	Severe (grade 3–4)	0 (0)	0		-
20th min	No shivering (grade 0)	24 (60.0)	16 (40.0)		-
	Mild (grade 1–2)	15 (37.5)	22 (55.0)	0.196	-
	Severe (grade 3–4)	1 (2.5)	2 (5.0)		-
25th min	No shivering (grade 0)	23 (57.5)	12 (30.0)		0.013 *
	Mild (grade 1–2)	14 (35.0)	19 (47.5)	0.027 *	0.256
	Severe (grade 3–4)	3 (7.5)	9 (22.5)		0.041 *
30th min	No shivering (grade 0)	24 (60.0)	10 (25.0)		0.002 *
	Mild (grade 1–2)	11 (27.5)	15 (37.5)	0.003 *	0.34
	Severe (grade 3–4)	5 (12.5)	15 (37.5)		0.010 *
60th min	No shivering (grade 0)	20 (50.0)	8 (20.0)		0.005*
	Mild (grade 1–2)	15 (37.5)	17 (42.5)	0.006 *	0.648
	Severe (grade 3–4)	5 (12.5)	15 (37.5)		0.010 *
Recovery bay	No shivering (grade 0)	21 (52.5)	9 (22.5)		0.006 *
	Mild (grade 1–2)	17 (42.5)	26 (65.0)	0.019 *	0.044 *
	Severe (grade 3–4)	2 (5.0)	5 (12.5)		0.235
Upon discharge	No shivering (grade 0)	26 (65.0)	15 (37.5)		-
	Mild (grade 1–2)	14 (35.0)	25 (62.5)	0.014 *	-
	Severe (grade 3–4)	0	0		-

Data to expressed as numbers (percentages); * statistically significant, *p* < 0.05.

## Data Availability

The data are available on request from the corresponding author. The data are not publicly available due to privacy issues.
